# A primate-specific short GluN2A-NMDA receptor isoform is expressed in the human brain

**DOI:** 10.1186/s13041-019-0485-9

**Published:** 2019-07-04

**Authors:** Hannah Warming, Chrysia-Maria Pegasiou, Aleksandra P. Pitera, Hanna Kariis, Steven D. Houghton, Ksenia Kurbatskaya, Aminul Ahmed, Paul Grundy, Girish Vajramani, Diederik Bulters, Xavier Altafaj, Katrin Deinhardt, Mariana Vargas-Caballero

**Affiliations:** 1 School of Biological Sciences, University of Southampton, University Road, Southampton, SO17 1BJ UK; 2 Wessex Neurological Centre, University Hospital Southampton, University of Southampton, Southampton, SO16 6YD, UK; 3 Neuropharmacology Unit, Bellvitge Biomedical Research Institute (IDIBELL), L’Hospitalet de Llobregat, Barcelona, Spain

**Keywords:** NMDA receptor, Synapses, Human, Primate, Resected, Neurosurgery, PSD-95, Glutamatergic

## Abstract

Glutamate receptors of the N-methyl-D-aspartate (NMDA) family are coincident detectors of pre- and postsynaptic activity, allowing Ca^2+^ influx into neurons. These properties are central to neurological disease mechanisms and are proposed to be the basis of associative learning and memory. In addition to the well-characterised canonical GluN2A NMDAR isoform, large-scale open reading frames in human tissues had suggested the expression of a primate-specific short GluN2A isoform referred to as GluN2A-S. Here, we confirm the expression of both GluN2A transcripts in human and primate but not rodent brain tissue, and show that they are translated to two corresponding GluN2A proteins present in human brain. Furthermore, we demonstrate that recombinant GluN2A-S co-assembles with the obligatory NMDAR subunit GluN1 to form functional NMDA receptors. These findings suggest a more complex NMDAR repertoire in human brain than previously thought.

## Introduction

NMDA receptors are activated by coincident glutamate binding and intracellular depolarisation. Ca^2+^ entry via NMDARs can gate long-term biochemical and gene expression changes that alter synaptic strength, which are proposed as central to mechanisms of memory storage [17] and neurodegenerative processes [9]. Our current knowledge of NMDAR function is largely derived from the study of rodent receptors and heterologous expression of cloned rodent genes. Tetrameric NMDARs comprise two obligatory GluN1 subunits and two GluN2 or GluN3 subunits, and in the adult forebrain GluN1/GluN2A, GluN1/GluN2B diheteromers, and GluN1/GluN2A/GluN2B triheteromers are the most common [18, 19]. The subunit combination confers the distinct biophysical and pharmacological properties to the receptor channel. The developmentally and anatomically regulated gene expression of NMDAR subunits, together with diverse post-translational modification mechanisms and protein interactions, determines the assembly, trafficking, synaptic or extrasynaptic localisation and internalisation of NMDARs (Reviewed in [16]) and their correct functioning is necessary for human brain functions [5, 6, 21].

**Fig. 1:**
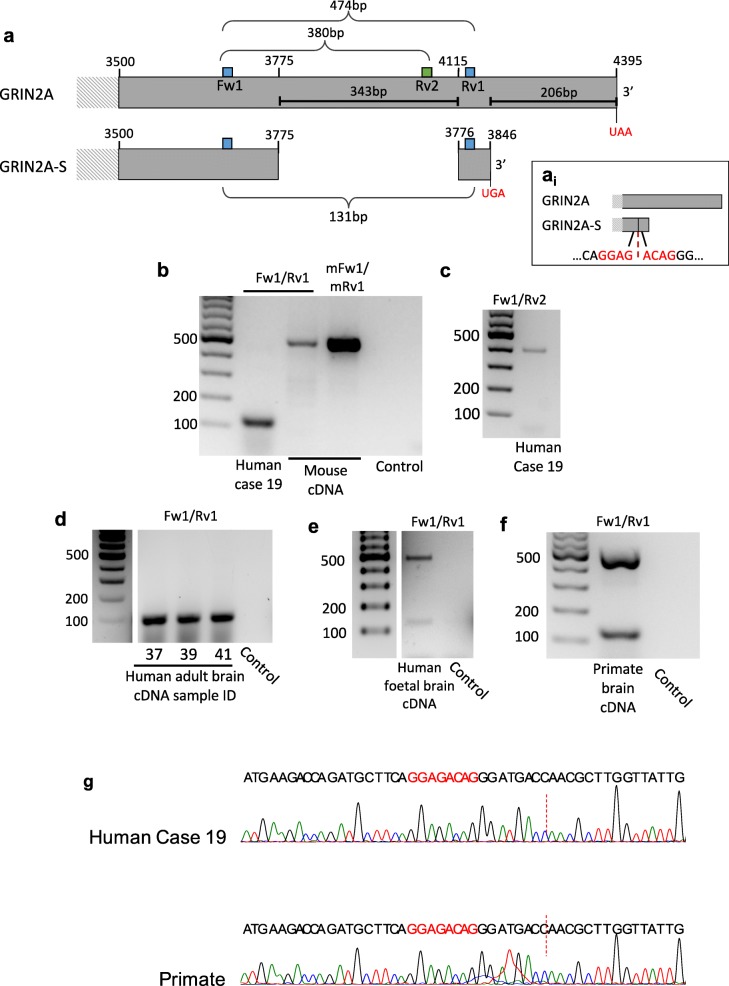
The *GRIN2A* gene has two transcript variants in human and primate but not mouse brain. **a** A short isoform of *GRIN2A* transcript was predicted by open reading frame studies. The published sequence of *GRIN2A-S* suggests that the final exon (exon 13) is missing two nucleotide regions compared to the canonical transcript: firstly, the lack of 343 nucleotides generates a putative exon 14 in *GRIN2A-S* (splice site shown in A_i_) and the final 206 nucleotides of the canonical form are lost altogether. We designed primers to amplify the region of variance between the two isoforms (Fig. 1, Fw1/Rv1) generating an amplicon of 474 bp in *GRIN2A* and 131 bp in *GRIN2A-S*. A second reverse primer was designed to amplify canonical *GRIN2A* selectively (Fig. 1a, Rv2) generating an amplicon of 380 bp. **b-f** RT-PCR amplification end products. Control conditions indicate no cDNA template was used in PCR **b** In human cDNA, only the short form of *GRIN2A* was observed likely due to preferential amplification in PCR, whereas only a long product of 474 bp was seen in mouse using either human or mouse specific primers. **c** The Fw1/Rv2 primer pair was used to confirm expression of canonical *GRIN2A* in the same human sample as shown in (B). **d** 3 other human cortical samples with *GRIN2A-S* amplified. **e** Both the short and long amplicons were observed in human foetal cDNA. **f** *GRIN2A-S* was observed in primate (Rhesus) brain cDNA. **g** Sequencing of human and primate RT-PCR short amplicons confirmed the presence of the putative splice site shown in Ai

Homologous rodent and human NMDARs do share highly conserved subunit sequences and exhibit almost identical pharmacological properties [10]. However, large scale open reading frame studies performed with mRNA from a mix of human tissues [20, 28] have suggested that in addition to the conserved NMDAR canonical isoform of GluN2A in chordates, a shorter isoform is produced in humans (GluN2A-S) generated by alternative splicing of human *GRIN2A* (Fig. 1a). Here, we show that this alternative NMDAR isoform is expressed in the human and primate brain, and that it forms functional receptors together with the obligatory subunit GluN1 [15]. The presence of alternative NMDAR subunits not expressed in rodent model systems indicates the existence of unexplored neural mechanisms in human synapses with relevance to normal function, ageing and neurological disease.

## Results and discussion

To test whether the GluN2A-S mRNA (*GRIN2A*) is expressed in human brain, we designed primers (Fw1/Rv1) flanking the region of exon 13 containing the 343 base pairs (bp) present only in *GRIN2A* (Fig. 1a). We predicted two distinct amplicons (474 bp and 131 bp) that would distinguish the *GRIN2A* and *GRIN2A-S* transcripts, respectively (Fig. 1a). Following PCR using cDNA from human brain (Table 1) as template, we observed the presence of a ~ 131 bp amplicon. In contrast, in mouse we observed one product of 474 bp, corresponding to the canonical isoform, using Fw1/Rv1 primers and a pair of primers modified to exactly match mouse *Grin2A* at the same location (mFw1/mRv1, Fig. 1b). If both short and long *GRIN2A* cDNAs are present in the human sample, the synthesis of shorter cDNA could overwhelm the early PCR cycles [27] and only generate the short amplicon. Using an additional *GRIN2A* specific reverse primer (Rv2), we confirmed the presence of canonical *GRIN2A* in this human cDNA sample (Fig. 1c). We observed the 131 bp band in further adult brain cDNAs tested with Fw1/Rv1 primer pair (Fig. 1d, two further samples not shown). Although its expression levels increase developmentally, GluN2A is expressed throughout the life course [2]. We tested foetal human brain cDNA and confirmed the expression of both *GRIN2A* and *GRIN2A-S* (Fig. 1e). Thus, our data confirm the presence of both canonical *GRIN2A* and the novel *GRIN2A-S* transcripts in human brain tissue samples.

**Table 1:** Details of resected human brain tissue samples

Case Number	Sex	Age	Reason for Surgery
004	M	52	Hippocampal sclerosis
007	F	21	DNET
008	M	71	Glioblastoma
010	M	28	Hippocampal sclerosis
011	F	42	Glioma
014	M	32	Hippocampal sclerosis
016	F	36	Hippocampal sclerosis
017	F	62	Hippocampal sclerosis
018	M	30	Cavernous malformation
019	M	19	Arteriovenous malformation
020	F	70	Arteriovenous malformation
021	M	49	Hippocampal sclerosis
022	F	58	Subarachnoid haemorrhage
024	F	50	Cavernous malformation
026	M	27	Mesial temporal DNET with signal changes in the hippocampus
028	F	38	Epilepsy
030	M	40	Cortical dysplasia
032	F	29	Tumour resection
037	M	52	Glioma
039	F	58	Tumour resection
041	M	41	Hippocampal sclerosis

*M* Male, *F* Female, *DNET* Dysembryoplastic neuroepithelial tumour

A BLAST search of the 131 bp sequence amplified by primers Fw1/Rv1 provided primate-specific predictive hits. To confirm whether *GRIN2A-S* transcript is present in primate brain, we tested Rhesus macaque brain cDNA with Fw1/Rv1 primers and confirmed the presence of *GRIN2A-S* (Fig. 1f). We sequenced the shorter PCR products for both the adult human and primate samples and confirmed the presence of the exact splice site reported in the European Nucleotide Archive (Coding: AAI17132.1; [20] (Fig. 1a,g).

**Fig. 2:**
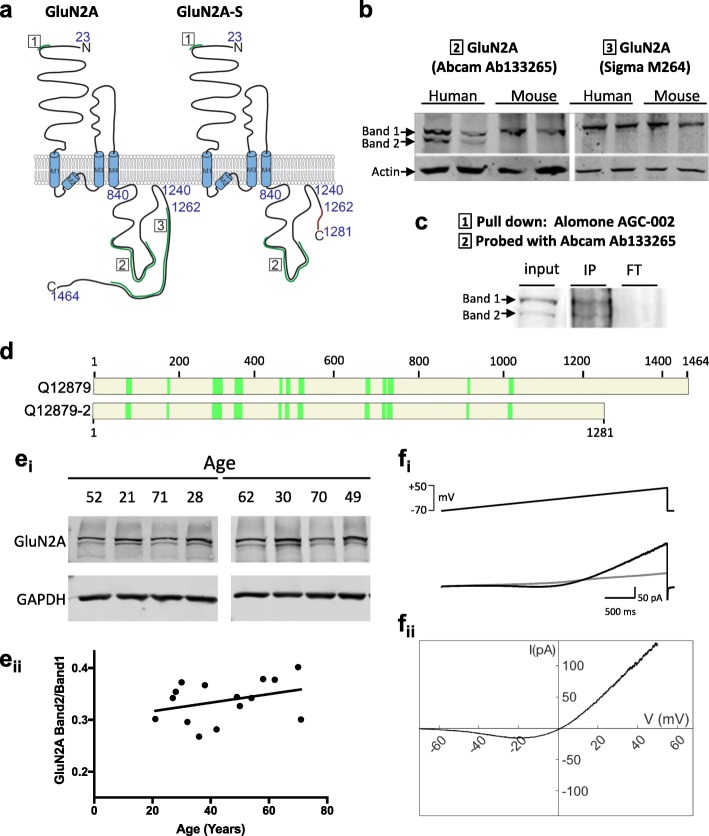
Two GluN2A protein bands are observed in human but not mouse brain. **a** Topology of the GluN2A subunit of the NMDA receptor and of GluN2A-S predicted from human mRNA studies. A spliced region is retained in canonical GluN2A leading to an alteration of the reading frame to generate a diverging C-terminal sequence (red) with early truncation. Epitopes for the antibodies used are shown in green and are numbered. **b** Immunoblots with specific antibodies against the canonical GluN2A and putative GluN2A-S protein in human and mouse cortical lysates. **c** GluN2A proteins were pulled down with an N-terminal antibody. Band 2 was cut from Coomassie-stained polyacrylamide gel and analysed by mass spectrometry. IP, immunoprecipitate. FT, flowthrough **d** A set of 14 peptides were confirmed to be present in this band. Figure shows tryptic peptides from band 2 coverage to either canonical GluN2A amino acid sequence (Uniprot: Q12879) or GluN2AS (Q12879–2, predicted) confirming band 2 contains GluN2A protein. **e** Homogenate from freshly frozen cortical human tissue probed with the GluN2A antibody Abcam 133,265 (select blots shown on top) and quantification of GluN2A-S / GluN2A immunoreactivity (bottom). See Table 1 for human tissue sample details. **f** Recombinant GluN2A-S co-expressed with GluN1 in HEK293 cells produces functional NMDARs as demonstrated by a typical J-shaped curve in response to 40 mM NMDA in response to a slow ramp of voltage (− 70 to + 50 mV, 3 s)

Furthermore, we aimed to evaluate whether the *GRIN2A* and *GRIN2A-S* transcripts were translated into the corresponding proteins. To this end, we hypothesised that if both transcripts are translated into proteins, two protein bands corresponding to GluN2A and GluN2A-S in human homogenate would be immunodetected by a GluN2A antibody targeting an epitope conserved between the canonical and short GluN2A isoforms (Fig. 2a,b). Importantly, Western blot analysis from human brain homogenates confirmed the presence of two immunoreactive bands with a molecular weight corresponding to the predicted GluN2A and GluN2A-S isoforms. On the contrary, a single immunoreactive band with the high molecular weight (corresponding to the canonical GluN2A) was detected in mouse brain lysates (Fig. 2b). Furthermore, using an antibody specifically detecting a carboxy terminal epitope (exclusively present in the canonical GluN2A isoform), we detected the presence of a single band, with a molecular weight corresponding to the canonical GluN2A subunit (Fig. 2a, b). To confirm the identity of the low molecular weight band, we immunoprecipitated GluN2A from human tissue homogenates with an antibody against the conserved N-terminal domain and analysed the primate-specific band (Band 2, Fig. 2c) by mass spectrometry. This unbiased method allowed the identification of 14 unique peptides located within GluN2A residues 81–1022, confirming that this low molecular band contains the proximal part of GluN2A, and thus discarding the potential cross-reactivity of anti-GluN2A N-terminal antibody (Fig. 2d). To assess the relative levels of GluN2A-S vs. total GluN2A, human cortical homogenates from fresh-frozen tissue resected from individuals 27–61 years of age were analysed by Western blot and the quantification showed that GluN2A-S immunoreactivity accounts for 34 ± 4% of canonical GluN2A protein in cortical human brain homogenate (Table 1) and this fraction remains constant across age (Fig. 2ei).

Finally, to test whether GluN2A-S can be incorporated into functional NMDARs, we co-transfected HEK293 cells with plasmids expressing GluN2A-S and GluN1 subunits [15]. A slow voltage ramp delivered during local perfusion with 40 μM NMDA and 10 μM glycine elicited a typical J-shaped curve (Fig. 2fi), and subtraction of responses without agonist (leak subtraction) revealed a typical NMDA current with reversal potential near 0 mV (Fig. 2eii). This confirms that GluN2A-S subunits are able to assemble with GluN1 subunits and become inserted into the plasma membrane to form a functional NMDAR that likely plays a role in human neural function.

Here we describe for the first time the brain expression of an uncharacterised, primate-specific NMDAR subunit. The splice site for GluN2A-S suggests that it will contain a diverging 19 aa sequence in its extreme C-terminal domain (Fig. 2a), in addition to lacking the distal carboxy terminal domain (183 amino acids) that contains PKC/SFK phosphorylation sites, two PDZ binding motifs that allow synaptic localisation [4, 12, 14], and a dileucin clathrin adaptor motif involved in receptor internalisation [13]. Following many lines of published evidence, these differences suggest that the dynamic regulation of GluN2A-S in response to stimuli could diverge from GluN2A subunit-containing NMDARs. This could impact the number of receptors present synaptically or extrasynaptically, the insertion of new receptors into the membrane, their lateral diffusion and clustering into synapses and their active removal. The potential changes in human synapses compared to mouse neurons void of GluN2A-S could result in distinct mechanisms involved in activity-dependent plasticity of synapses, which will highly depend upon its triheteromeric partners [1, 8, 19].

Together, our data suggest that GluN2A-S is a primate-specific NMDAR subunit and a substantial component of functional NMDARs in the adult human brain. Many neuronal mechanisms discovered in mice have been successfully recapitulated in humans. However, mounting evidence suggests that there are important differences between rodent and human neurons that result in distinct signal integration properties [22, 23, 26] and proteomic composition of synapses [3]. Species differences may ultimately impact the way in which human neural circuits can be computationally modelled [7], and the translation of pre-clinical findings into approved therapies [24]. Further analyses of GluN2A-S spatio-temporal gene expression and synaptic/ extrasynaptic localisation will enhance our knowledge of their functional role and may uncover NMDAR trafficking mechanisms present only in primates and diverging sequences may uncover novel therapeutic targets.

## Materials and methods

### Human brain tissue samples

All samples consisted of cortical tissue resected for access to deeper brain lesions such as sclerotic hippocampus in epileptic patients (the pathological tissue for these lesions was not used). Informed consent was obtained from all patients to use surgically-resected putatively non-pathological tissue not required for diagnostic purposes (see ethical approval declaration). Briefly, resected tissue was obtained from temporal cortex of patients undergoing surgery for the removal of deeper structures. Tissue was collected in ice cold artificial cerebrospinal fluid [26] then taken to the laboratory, frozen and kept at − 80 °C. Transfer time was of the order of 10–15 min.

### Mouse brain tissue

Mice were decapitated following isoflurane anaesthesia (see ethical approval declaration). Brains were extracted in ice-cold ACSF and sliced or snap-frozen. All brain tissue samples were stored in the -80C freezer until lysed.

### RNA extraction and cDNA synthesis

Total RNA was isolated from human and mouse tissue using Trizol and then purified using the RNeasy Mini kit (QIAGEN) following the manufacturer’s instructions. cDNA was synthesised immediately from 200 ng of total RNA per reaction using the SuperScript IV reverse transcriptase and cDNA synthesis kit (INVITROGEN) according to the manufacturer’s instructions. The cDNA obtained was stored at − 80 °C. Human foetal cDNA was obtained from Takara (Normal brain tissue cDNA, pooled from 59 spontaneously aborted male/female Caucasian fetuses, ages: 20–33 weeks). Rhesus macaque cDNA was obtained from Amsbio (Normal brain tissue, Female, 4.5 years).

### PCR conditions and primers

PCR was performed on 1μg of cDNA using primers and REDTaq® Readymix™ PCR Reaction Mix (Sigma-Aldrich) for 40 cycles. DNA was denatured at 95 degree C and extended at 72 degree C for 45 s each cycle. Products were separated on a 1.5% agarose gel.

The following primers were used: Fw1: ATTCAGGCCACTTCACCATGAG, Rv1: ATCTCCCAATAACCAAGCGTTG, Rv2: CTTGCTGTCCTCCAGACCTTGG mFw1: ACTCAGGCCACTTTACCATGAG, and mRv1: ATCTCCCAATAACTAAGCGTTG.

### Plasmids

pEYFP-NR1a was a gift from Stefano Vicini (Addgene plasmid # 17928; [15]). *GRIN2A-S* plasmid from Broad Institute, was acquired from Source Bioscience (Transcript NM_001134408.2).

### Western blotting

Equal amounts of protein (28 μg) were separated in 7.5% acrylamide gels by SDS-PAGE and transferred onto nitrocellulose membranes. Membranes were blocked in 5% (w/v) non-fat milk for 1 h at room temperature and incubated overnight at 4 C in 5% (w/v) bovine serum albumin (BSA) containing 0.1% (v/v) Tween-20 and one of the following primary antibodies: anti-NMDAR2A (ab133265; 1:1000; Abcam); and GAPDH (D16H11; 1:1000; CST) actin. Membranes were washed 3 times with Tris-buffered saline (TBS) containing 0.1% Tween-20 (TBS-T) and probed with fluorophore-conjugated goat anti-mouse/−rabbit secondary antibody (1:10000; LI-COR). Proteins were visualised using the Odyssey infrared scanner (LI-COR) using Image Studio Light Software.

### GluN2A pulldown

GluN2A was pulled down from 1 mg of protein homogenate (in RIPA buffer) using 2 μg of Alomone antibody AGG-002 beads. Eluate was run in SDS page and stained with Coomassie dye. The lighter band corresponding to putative GluN2A-S was cut and analysed by LC-MS/MS [11].

### HEK293T cell recordings

HEK293T cells were cultured at 37 C with 5% CO_2_ in Dulbecco’s Modified Eagle Medium with glucose, L-glutamine and pyruvate, 10% FBS and 1% Pen-Strep and seeded at low density onto poly-L-lysine coated glass coverslips for electrophysiology. Adherent cells were transfected using JetPEI reagent with NR1a and GluN2A-S plasmids at a 1:1 ratio and recorded after 48 h. Borosilicate glass micropipettes were pulled to produce a resistance of 4–6 mOhm and filled with intracellular recording solution containing in mM: Gluconic acid 70, Caesium chloride 10, sodium chloride 5, BAPTA 10, HEPES 10, GTP 0.3 ATP 4 and pH balanced to 7.3 with caesium hydroxide. Cells were perfused with aCSF throughout recording containing, in mM: sodium chloride 126, calcium dichloride 2, glucose 10, magnesium sulfate 2, potassium chloride 3, NaH_2_PO_4_ 1.25 and NaHCO_3_ 26.4, and glycine 10 μM and pH regulated by continuous bubbling of 95% oxygen, 5% CO_2_. Recordings with or without addition of NMDA 40 μM were made in whole-cell voltage clamp and Matlab software and amplified using an Axopatch 200B as previously described [28].

## Data Availability

The datasets used and/or analysed during the current study are available from the corresponding author on reasonable request.

## Abbreviations

bp: Base pairs
cDNA: Copy deoxyribose nucleic acid
GAPDH: Glyceraldehyde 3-phosphate dehydrogenase
LC-MS: Liquid chromatography – mass spectrometry
mRNA: Messenger ribonucleic acid
NMDA: N-methyl-D-aspartate
NMDAR: N-methyl-D-aspartate receptor
PCR: Polymerase chain reaction
SDS-PAGE: Sodium dodecyl sulfate polyacrylamide gel electrophoresis
